# Polystyrene Degradation by *Exiguobacterium* sp. RIT 594: Preliminary Evidence for a Pathway Containing an Atypical Oxygenase

**DOI:** 10.3390/microorganisms10081619

**Published:** 2022-08-10

**Authors:** Anutthaman Parthasarathy, Renata Rezende Miranda, Nathan C. Eddingsaas, Jonathan Chu, Ian M. Freezman, Anna C. Tyler, André O. Hudson

**Affiliations:** 1 Thomas H. Gosnell School of Life Sciences, Rochester Institute of Technology, Rochester, NY 14623, USA; 2 School of Chemistry and Biosciences, University of Bradford, Bradford BD7 1DP, UK; 3 School of Chemistry and Materials Science, Rochester Institute of Technology, Rochester, NY 14623, USA

**Keywords:** *Exiguobacterium*, polystyrene, biodegradation, biotransformation, oxygenase, microbial metabolism, plastic degradation

## Abstract

The widespread use of plastics has led to their increasing presence in the environment and subsequent pollution. Some microorganisms degrade plastics in natural ecosystems and the associated metabolic pathways can be studied to understand the degradation mechanisms. Polystyrene (PS) is one of the more recalcitrant plastic polymers that is degraded by only a few bacteria. *Exiguobacterium* is a genus of Gram-positive poly-extremophilic bacteria known to degrade PS, thus being of biotechnological interest, but its biochemical mechanisms of degradation have not yet been elucidated. Based solely on genome annotation, we initially proposed PS degradation by *Exiguobacterium* sp. RIT 594 via depolymerization and epoxidation catalyzed by a ring epoxidase. However, Fourier transform infrared (FTIR) spectroscopy analysis revealed an increase of carboxyl and hydroxyl groups with biodegradation, as well as of unconjugated C-C double bonds, both consistent with dearomatization of the styrene ring. This excludes any aerobic pathways involving side chain epoxidation and/or hydroxylation. Subsequent experiments confirmed that molecular oxygen is critical to PS degradation by RIT 594 because degradation ceased under oxygen-deprived conditions. Our studies suggest that styrene breakdown by this bacterium occurs via the sequential action of two enzymes encoded in the genome: an orphan aromatic ring-cleaving dioxygenase and a hydrolase.

## 1. Introduction

Plastic degradation is one of the major environmental concerns of the 21^st^ century that requires urgent solutions. Several bacteria and fungi are known to degrade synthetic polymers (plastics) in the natural environment. Among the known bacterial strains, *Ideonella sakaensis* can degrade polyethylene terephthalate (PET) [1] and several *Pseudomonas* species degrade a variety of polymers, including polyethylene (PE), polypropylene (PP), polyvinyl chloride (PVC), polystyrene (PS), polyurethane (PU), PET, polyethylene succinate (PES), polyethylene glycol (PEG), and polyvinyl alcohol (PVA) [2]. In addition, low-density polyethylene (LDPE) has been shown to be degraded by bacteria residing in the guts of earthworms [3]. Of all the plastics undergoing microbial degradation, PET can be degraded by the widest variety of enzymes: 19 types of hydrolases belonging to the esterase, lipase, and cutinase enzyme families [4,5,6]. Therefore, the use of microbe-based technologies offers a feasible and sustainable strategy to address the issue of plastic accumulation and pollution [7]. Furthermore, biotechnological platforms can provide “green” approaches to derive monomers from waste and pollutants to facilitate the transition to a more circular economy [8]. In fact, the use of machine learning has enabled the construction of a mutant PET-degrading enzyme called FAST-PETase with superior activity compared to the wild type, and subsequent repolymerization of the recovered monomers into PET has demonstrated the possibility of a closed-loop recycling involving enzymatic depolymerization and chemical repolymerization [9].

Among the various plastic polymers, PS is considered a recalcitrant type [10]. PS depolymerization activity may be conferred by depolymerase enzymes whose substrates are long-chain alkanes or aromatic compounds [11]. For example, it has been suggested that aromatic ring hydroxylases may be involved in the depolymerization of higher molecular weight styrene units into styrene [10]. Styrene can be converted into intermediates of the tricarboxylic acid (TCA) cycle by several bacterial genera, such as *Pseudomonas*, *Nocardia*, *Xanthobacter*, and *Rhodococcus* [12]. Tischler and collaborators discovered a side-chain oxygenation pathway in *Rhodococcus opacus* 1CP that converts styrene to styrene oxide via styrene monooxygenase (SMO), followed by isomerization to phenylacetaldehyde by the action of styrene oxide isomerase (SOI) [13]. Additional chemical transformations of styrene include reduction to ethylbenzene, side chain hydroxylation to either 1-phenylethanol or 2-phenylethanol [14], and conversion to phenylacetic or benzoic acids via anaerobic degradation [15,16]. *Rhodococcus rhodochrous* NCIMB 13259 can cleave styrene via ring dioxygenation into vinylcatechol, which is further degraded into acetaldehyde and pyruvate [17]. 

PS has also been converted to styrene oil via pyrolysis, and the resulting product can be converted by *Pseudomonas putida* CA-3 into the storage biopolymer polyhydroxyalkanoate (PHA) [18]. *Cupriavidus necator* H16 is another bacterium that converts PS into PHA [19]. PS films were reported to be superficially oxidized by the brown rot fungus *Gloeophyllum trabeum* [20]. The genus *Bacillus* contains strains able to degrade brominated high impact PS [21], as well as *Bacillus cereus* and *Bacillus gotheilii*, which degrade PS granules [22]. Darkling beetles (*Plesiophthalmus davidis*) in the larval stage can degrade PS in the form of Styrofoam, and *Serratia* sp. was the PS-degrading gut bacterium in this case.

Bacteria belonging to the genus *Exiguobacterium* have also demonstrated the ability to degrade PS. Yellow mealworms (the larval form of *Tenebrio molitor* Linn.) can degrade PS based on its gut microbiome, whereby the bacterium conferring the PS-degradation capacity is a strain of *Exiguobacterium* sp. [11,23]. Biofilms of two other *Exiguobacterium* strains, DR11 and DR14, have also been shown to degrade PS [24]. The genus *Exiguobacterium* contains non-spore forming poly-extremophiles that tolerate freezing, heat, boiling water, desiccation, heavy metals, alkaline pH, pesticides, dyes, organic solvents, and UV radiation [25,26,27,28,29,30,31,32,33,34,35,36,37,38,39,40,41,42,43,44,45,46,47,48,49]. These characteristics and the fact that genetic modification of this genus is possible [50] make *Exiguobacterium* sp. an attractive platform for developing PS-degradation biotechnology. Although previous studies have shown that degradation of PS involves mainly the generation of -COOH and -OH groups by the *Exiguobacterium* sp. [11,23,24], the biochemical mechanisms remain nebulous. Nevertheless, the enzymes expressed specifically by *Exiguobacterium* sp. have not been examined. Instead, several enzymes, such as β-galactosidase, acid phosphatase, β-glucuronidase, naphthol-AS-BI-phosphohydrolase, leucine arylamidase, and alkaline phosphatase, were detected in the gut bacterial community of *T. molitor* (which includes *Exigobacterium* sp.) [51], but the PS degradation pathway was not elucidated in this case.

## 2. Materials and Methods

### 2.1. Media, Chemicals, Bacterial Strains, and Consumables

Bacterial culture media were bought from Merck, agar and agarose from Fluka, primers from Invitrogen, sterile filters (0.4 and 0.2 μm) from Corning Inc. (Corning, NY, USA), and polystyrene sheets (0.19 mm thickness) from Goodfellow Cambridge Ltd., Huntingdon, UK. All other chemicals were bought from Merck, Kenilworth, NJ, USA, and Sigma-Aldrich, St. Louis, MI, USA, unless mentioned otherwise.

### 2.2. Microbial Culture and Isolation of Strains

Soil samples were collected from various sites around the campus of the Rochester Institute of Technology (RIT), Rochester, NY, USA, with the help of a sterile hand shovel washed with 70% ethanol and stored in sterile 50 mL Falcon tubes. A total of 1 g of soil was suspended in 5 mL of sterile LB medium and grown overnight at 30 °C with shaking at 200 rpm. The cultures were spun at 3000× *g* for 5 min on a tabletop centrifuge to sediment the soil particles. Clear culture free of soil particles was pipetted, serially diluted (10^−1^ to 10^−10^), plated on LB agar plates, and grown overnight at 30 °C. After visual observation of the bacterial diversity based on size, color, shape, morphology, and texture, colonies were selected and re-streaked on fresh LB plates to grow overnight at 30 °C. The re-streaking process was repeated until a pure culture was obtained. The strain reported in this study originated from the rhizosphere of an oak tree on a managed landscape at the RIT campus.

### 2.3. PCR Amplification and Nucleotide Sequencing of the 16S V3–V4 Regions

Colony PCR was performed using the primers 341F (5′-CCTACGGGNGGCWGCAG-3′) and 805R (5′-GACTACHVGGGTATCTAATCC-3′) for the V3–V4 region using the GoTaq Green Mix containing the GreenTaq polymerase from Genescript Inc. PCR conditions included denaturation at 95 °C for 5 min, followed by 30 cycles at 94 °C for 1 min, 50 °C for 1 min (annealing), and 72 °C for 1 min, with a final extension at 72 °C for 10 min. The amplified products were visualized by electrophoresis on a 1% agarose gel pre-stained with ethidium bromide, run at 70 V for 45 min, and using 1× TAE as the running buffer in a model 250 electrophoresis chamber (Life Technologies, Carlsbad, CA, USA). The 1 kb Plus DNA ladder (Invitrogen, Waltham, MA, USA) was added as a standard. The amplified products were purified using a PCR cleanup kit (Bio Basic Canada Inc., Markham, ON, Canada), quantified with a Nanodrop One microvolume UV-visible spectrophotometer (Thermo Scientific, Waltham, MA, USA), and Sanger sequencing with 341F as the sequencing primer at the Genewiz sequencing facility, South Plainfield, NJ, USA. The generated sequences were compared using the Basic Local Alignment Search Tool (blastn) [52] to identify the genera using the default parameters. Further taxonomic analysis based on the full 16S sequence and whole genome analysis was performed as described in Section 2.4.

### 2.4. Whole Genome Sequencing, Strain Identification, and Other Bioinformatics Analysis

The initial genus and species identification of *Exiguobacterium* sp. RIT 594 was based on the amplification and sequencing of the V3–V4 16S rRNA gene. This was further confirmed using the entire 16S rRNA gene sequence derived from the whole genome, which was facilitated by whole-genome sequencing using the Illumina MiSeq platform following a reported procedure [53]. The genome of *Exiguobacterium* sp. RIT 594 has been submitted to GenBank under the accession number QPKF00000000.

The assembled FASTA contig files for *Exiguobacterium* sp. RIT 594 was uploaded to Type Strain Genome Server (TYGS) found at https://tygs.dsmz.de (accessed on 15 July 2022). TYGS is a freely available tool for creating taxonomic assignments based on whole-genome sequence data [54,55]. TYGS infers trees with FastME 2.1.4 based on Genome BLAST Distance Phylogeny (GBDP) distances calculated from the genome sequences. Branch lengths are scaled in terms of GBDP distance formula d5 [56]. We have used all of the other (fifteen) available *Exiguobacterium* genomes publicly available for comparison to enable whole genome-based taxonomic assignment. The genome in the GenBank entry was accessed and scanned for proteins with the search terms oxygenase, monooxygenase, dioxygenase, and hydrolase. The entries returned for *Exiguobacterium* sp. RIT 594 were compared to known PS degradation enzymes using the protein BLAST function with default parameters [52]. Multiple sequence alignments of the amino acid sequences were performed using Clustal Omega [57].

### 2.5. Detection of Polymer Degradation

The strains were tested by culturing in 30 mL of LB medium in 50 mL Falcon tubes at 30 °C overnight (16 h) and adding sterilized polystyrene (1 × 1 cm squares of polystyrene; thickness 0.19 mm), which were cut, unless otherwise mentioned (some of the older samples were 2 × 3 cm) and sterilized in 100% ethanol for 20 min. Polystyrene squares were placed into cultures using sterile tweezers. Control tubes were made with 30 mL of growth media. Experimental and control samples were incubated at 30 °C at 100 rpm for a desired period. For the oxygen-deprived cultures, Falcon tube lids were screwed on tight and sealed with duct tape, but shaking was performed at 100 rpm to allow for homogenous mixing. The period in which oxygen becomes limited is uncertain, but due to the consumption of oxygen in rich media and the poor solubility of oxygen in water, sealed cultures more than 14 days old are expected to be oxygen deprived.

Following incubation over the desired growth period, the cells were centrifuged at 4000× *g* for 20 min in a VWR Clinical 200 centrifuge, and the polymer pieces were retrieved from the cultures with a sterilized tweezer. After visual inspection, the polymer containing bacterial biofilms were prepared for electron microscopy [58]. Attempts were also made to cultivate the strains under the same temperature and shaking conditions for 60 days with sterilized polystyrene in liquid carbon-free basal medium (LCFBM), which has the following composition (per L): 0.7 g KH_2_PO_4_, 0.7 g K_2_HPO_4_, 0.7 g MgSO_4_·7H_2_O, 1.0 g NH_4_NO_3_, 0.005 g NaCl, 0.002 FeSO_4_·7H_2_O, 0.002 g ZnSO_4_·7H_2_O, and 0.001 g MnSO_4_·H_2_O. This medium is the recommended American Society for Testing and Materials (ASTM) standard for determining the resistance of plastics to bacteria [59].

### 2.6. Scanning Electron Microscopy (SEM)

Samples for SEM analysis were prepared following a published procedure [58]. The polymer pieces covered with biofilm were rinsed gently with phosphate buffered saline (PBS) to remove non-adherent cells. The films were soaked in 2% glutaraldehyde in (PBS) buffer pH 7.4 for fixation of cells for 2 h at room temperature. The samples were rinsed 3 × 5 min using the same fixative buffer. Next, the samples were dehydrated in 50–80% graded ethanol for 10 min each (steps of 50%, 70%, and 80%), followed by 2 × 5 min rinses with 95% ethanol, and 3 × 15 min rinses with fresh 100% ethanol. All liquid was removed by pipetting and the samples were stored (sealed with Parafilm) at 4 °C overnight. Prior to SEM, the samples were coated for 2 min with gold-palladium using an SPI sputter coater to mitigate charging in the electron beam. SEM was performed at a voltage of 5 kV using a Mira3 Tescan field-emission SEM from the Nanoimaging Lab at the Rochester Institute of Technology.

### 2.7. Fourier Transform Infrared (FTIR) Spectroscopy

Surface degradation was analyzed by attenuated total reflectance (ATR)-FTIR spectroscopy using Shimadzu IRTracer-100 (Kyoto, Japan) with a QATR 10 ATR attachment (diamond crystal) and an MCT detector. All samples were scanned from 4000 to 600 cm^−1^ at a resolution of 4 cm^−1^, with 16 scans averaged. The penetration depth into the samples using a diamond ATR crystal is ~2 µm, so spectra obtained are of the surface. The low penetration depth allowed for separate analysis of polystyrene present at both the top, exposed to the bacterium, and the bottom of the petri dish. This provided conformation that the spectral changes were due bacterial degradation.

## 3. Results and Discussion

### 3.1. Strain Identification Using 16S rRNA and Whole Genome-Based Phylogeny

**Figure 1:**
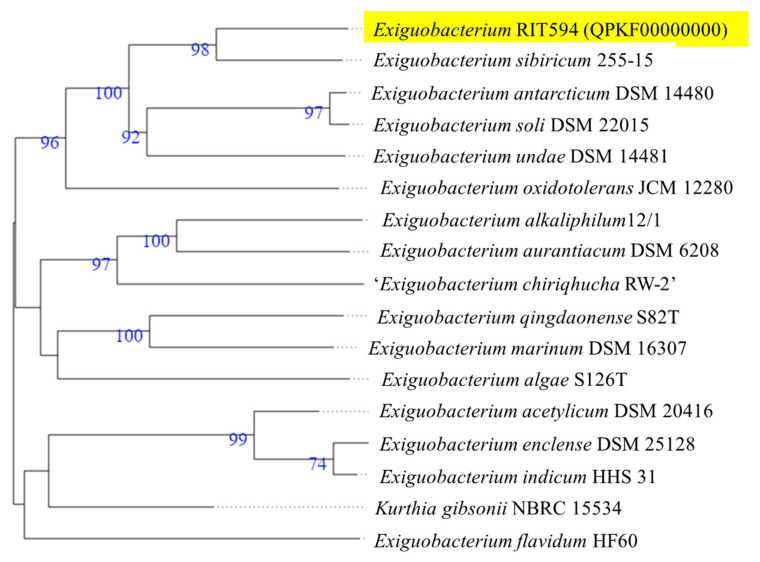
Whole genome-based phylogeny of the *Exiguobacterium* sp. RIT 594 isolate predicted by TYGS (see earlier references) shows the closest relative to be *Exiguobacterium sibiricum* 255-15. TYGS analysis is based on Genome BLAST Distance Phylogeny (GBDP) with distances calculated from the genome sequences. Branch lengths are scaled in terms of GBDP distance formula and the numbers above the branches are GBDP pseudo-bootstrap support values from 100 replications.

16S (full gene derived from the genome sequence) and whole genome-based phylogenetic analysis of the RIT 594 strain based on TYGS confirmed it as *Exiguobacterium* sp., with the nearest relative being *Exiguobacterium sibiricum* 255-15. The whole genome-based phylogenetic tree comparing RIT 594 to all other *Exiguobacterium* genomes deposited to date as references is shown in Figure 1.

### 3.2. Detection of Polystyrene (PS) Degradation

**Figure 2:**
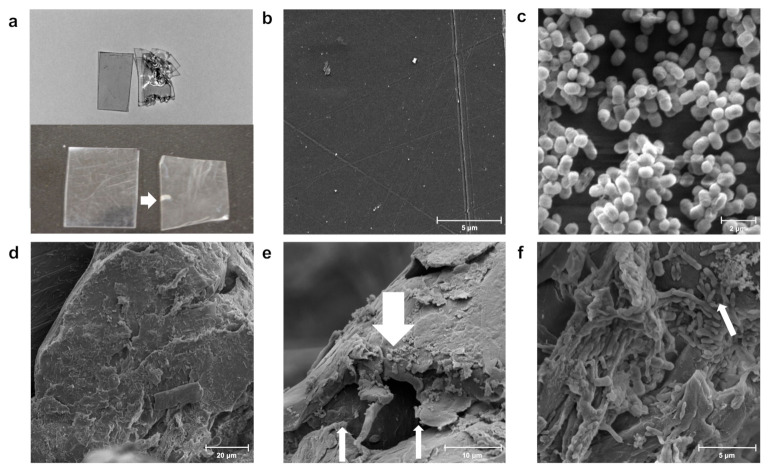
Macroscopic and microscopic images of polystyrene (PS) degradation by *Exiguobacterium* sp. RIT 594. (**a**) Macroscopic images of circa 2 × 3 cm pieces cut from PS plates (0.19 mm thickness); top left and bottom left = sterile control; top right and bottom right = incubated in shaking cultures of *Exiguobacterium* sp. RIT 594. The samples were incubated for 21 (top) or 35 (bottom) days. The pattern of degradation varies with each sample: the top one shows visible holes, while the bottom one shows opaque notches indicated by a white arrow. (**b**–**f**) SEM images with resolutions indicated. (**b**) PS control without bacterium (11,700× resolution, scale bar 5 μm); (**c**) pure cultures of *Exiguobacterium* sp. RIT 594 grown in liquid media (LB) without PS and deposited on a cover slip (16,000× resolution, scale bar 2 μm) are seen as cocci. (**d**–**f**) Increasing magnification of the white notch from the plate on bottom right in (**a**) at (**d**) 1990× resolution, scale bar 20 μm; (**e**) 5240× resolution, scale bar 10 μm (note the areas where the polymer is eaten away as indicated by the thinner arrows and the bacterium on the polymer by thicker arrows); and (**f**) 10,300× resolution, scale bar 5 μm, where the white arrow points to the shortened rods in the biofilm with characteristic fibers and nanoparticles generated during degradation.

The goal of our study was to identify bacterial strains that can degrade plastics from our strain collection. We chose PS since it is recalcitrant and because there were prior reports of *Exiguobacterium* sp. degrading this polymer [10,21,22]. We selected the bacterium *Exiguobacterium* sp. RIT 594 from a cohort of 12 strains of various genera because a macroscopic (naked eye) examination of thin PS sheets colonized by this strain for 21–35 days in LB medium (Figure 2a) showed various degradation patterns, such as millimeter-sized holes, wrinkling, or opaque notches on the plastic surface. We associated degradation with the presence of the bacterium since notches, wrinkles, or holes were not observed on virgin PS (Figure 2b). Presumably, the bacterium first migrated to rough patches or microscopic defects on the polymer surface, formed biofilms, and then expanded to rest of the polymer. We then performed scanning electron microscopy (SEM) with either culture of *Exiguobacterium* sp. RIT 594 in rich medium (LB), or controls for both PS and bacterium, to better visualize the bacterial activity on PS surfaces. A control sample grown in broth culture without PS and deposited on a glass cover slip revealed short, rounded cocci of *Exiguobacterium* sp. RIT 594 about 0.5 μm wide and less than 1 μm long (Figure 2c). An examination of an opaque notch seen in the 35-day old sample (Figure 2a) revealed significant degradation of the polymer (Figure 2d–f) as opposed to the uninoculated control (Figure 2b) over 35 days.

**Figure 3:**
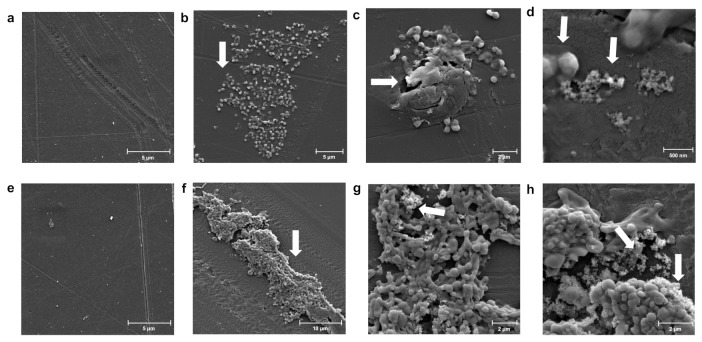
Biofilm formation by *Exiguobacterium* sp. RIT 594 on PS over 14 days. The pattern of distribution of the particles, both inside the biofilm as well as in contact with the cells, suggests that these particles arise from the active biological degradation of PS; the most interesting features of the SEM images are indicated by white arrows. (**a**) A control PS piece of 0.19 mm thickness at 11,700× resolution, scale bar 5 μm; (**b**–**d**) *Exiguobacterium* sp. RIT 594 grown in LB with sterilized PS pieces for 7 days. (**b**) 7250× resolution, scale bar 5 μm, showing a thin biofilm; (**c**) 16,000× resolution, scale bar 2 μm, showing “eruptions” on the polymer surface containing biofilm associated with groups of smaller particles; (**d**) 79,000× resolution, scale bar 0.5 μm, showing nanometer-sized particles associated with cells; (**e**) a control PS piece of 0.19 mm thickness at 11,700× resolution, scale bar 5 μm. (**f**–**h**) *Exiguobacterium* sp. RIT 594 grown in LB with sterilized PS pieces for 14 days. (**f**) 5700× resolution, scale bar 10 μm, showing a thicker biofilm; (**g**) 17,200× resolution, scale bar 2 μm, showing cells in a biofilm associated with nanometer-sized particles; (**h**) snapshot of (**g**) at 23,400× resolution, scale bar 2 μm.

Next, we investigated if the biodegradation of PS by *Exiguobacterium* sp. RIT 594 would produce any microscopic plastic particles, since earlier studies showed that nanometer scale particles can be released from the parent polymer because of partial initial degradation via abiotic weathering [60]. We observed the development of a thicker biofilm over a time frame of 14 days (Figure 3), as well as a pattern of distribution of the nanometer sized polymer particles within the biofilm and on the cell surface. The nanometer sized fibers or particles were not seen in the negative controls that lacked the bacterium but contained PS, strongly indicating that biological degradation of PS generated these nanoparticles and that, under the conditions we used, abiotic weathering is not a strong contributor. Growth medium was ruled out as the controls did not contain the nanoparticles, and bacterial nutrient starvation is unlikely since the bacterium was in LB (rich medium).

### 3.3. Changes in Morphology during Colonization of Different Polymers

**Figure 4:**
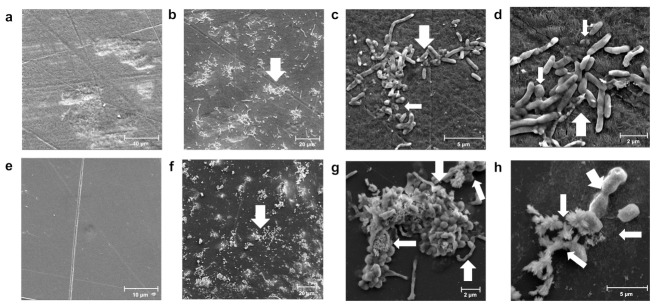
Colonization of different polymers by *Exiguobacterium* sp. RIT 594. The surfaces of high-density polyethylene (HDPE) (**a**–**d**) and of PS (**e**–**h**), were recorded following 60 days of shaking in minimal medium (LCFBM) lacking a carbon source other than the polymer. (**a**) Control without bacterial inoculation (4980× resolution, scale bar 10 μm); (**b**–**d**) colonized HDPE by *Exiguobacterium* sp. RIT 594. (**b**) Colonies of the bacterium at 1760× resolution and scale bar at 20 μm (white arrow); (**c**,**d**) represent snapshots of (**b**) at magnifications of 11,200 and 20,300×, and scale bars at 5 and 2 μm, respectively. The typical short, rounded morphology is highlighted with thinner white arrows, and the elongated rods with thicker white arrows. The cell shape is possibly elongated due to the inability to use HDPE as a carbon source (starvation response; see text for details). (**e**) Control without bacterial inoculation (5000× resolution, scale bar 10 μm), 60 days shaking in minimal medium (LCFBM), and (**f**–**h**) colonized by *Exiguobacterium* sp. RIT 594. (**f**) Colonies of the bacterium at 1400× resolution and scale bar at 20 μm (white arrow). (**g**) Further magnification of (**f**) reveals that the cells are associated with nanoscale fibers and particles (thinner white arrows) and cell elongation (thicker white arrows); 13,700× resolution, scale bar 2 μm. (**h**) Multiple cells adhere to a cell with a single long projection (thicker white arrow), which is associated with nanoscale fibers and particles (thinner white arrows; 29,500× resolution, scale bar 2 μm).

We then compared the efficiencies and patterns of biodegradation when the bacterium was incubated with different plastic polymers. *Exiguobacterium* sp. RIT 594 was able to colonize high-density polyethylene (HDPE) when grown in liquid carbon-free basal medium (LCFBM), a minimal medium lacking extra carbon sources besides the polymer itself. Figure 4a shows an uninoculated control. Nanoscale fibers or particles of the polymer were not observed in this case (Figure 4b–d), whereas cell elongation was, which could be due to nutrient limitation, leading to a starvation response. Conversely, *Exiguobacterium* sp. RIT 594 appeared to degrade PS when grown in LCFBM since nanoscale fibers and particles were seen associated with the cells (Figure 4f–h). *Exiguobacterium* strains have been previously reported to nearly triple their cell length after exposure to organic solvents [61]. Cell elongation in response to different stresses by different strains in the *Exiguobacterium* genus suggests that it could be a generic stress response. Although some elongation can be observed in *Exiguobacterium* sp. RIT 594 when PS is added to the culture in a minimal medium for 60 days, the starvation physiology (notable fattening and elongation) seen with HDPE is not observed. Therefore, we inferred that the bacterium may potentially utilize PS as a sole carbon source, even though the growth is very slow and difficult to quantify. For comparison, PS degradation proceeds to a significant extent only after 28 days, even in LB medium.

**Figure 5:**
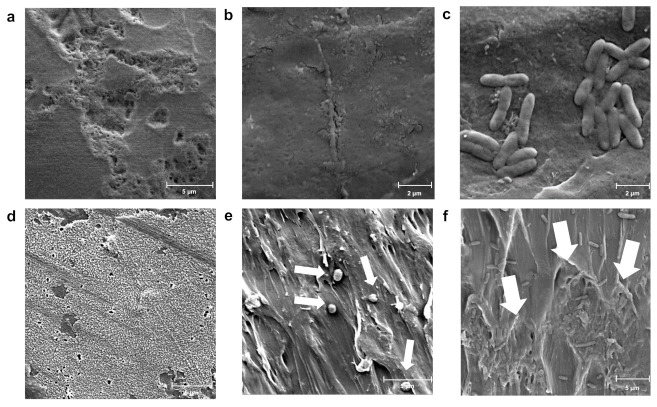
Styrofoam degradation by *Exiguobacterium* sp. RIT 594. PS was obtained directly from Styrofoam coffee cups (US size small) for (**a**–**c**). (**a**) Uninoculated control (9930× resolution, scale bar 5 μm). (**b**,**c**) 35-day old cultures containing Styrofoam pieces (17,700× resolution, scale bar 2 μm, and 17,700× resolution, scale bar 2 μm, respectively). Note how the biofilm is sparse in contrast to earlier examples and in the cells seen in Figure 2c,d,g,h; (**c**) cells are fattened and elongated, suggesting starvation. (**d**–**f**) PS in the form of transparent flakes dissolved in toluene and dried inside a fume hood to remove the solvent. (**d**) An uninoculated control; (**e**,**f**) 35-day old cultures containing PS flakes. Cells are either embedded on the surface as cocci (**e**) or congregate as short rods inside the polymer (**f**) (see white arrows), a morphology also seen in Figure 4h. (**d**–**f**) Resolutions are 8790, 10,300, and 7350×, respectively, and the scale bars are 5 μm.

The colonization of PS and the extent of its degradation was seen to depend on the surface properties of the type of PS used under identical experimental conditions. When Styrofoam from a typical small US coffee cup was used (Figure 5a–c), the cells did not readily attack the polymer, but rather started showing cell thickening and elongation characteristics seen during starvation. We believe this happened due to the lower wettability of the PS caused by waxy additives in Styrofoam. To further test this hypothesis, we dissolved Styrofoam in toluene, evaporated the organic solvent, and incubated the bacterium with the resulting dried flakes (Figure 5d–f). We observed that the cells were able to colonize and degrade PS under these conditions. Moreover, the cellular morphology was round when the cells were on the PS surface, and rod-like when they were inside a biofilm on the PS, which is a trend that can also be observed in Figure 3. Spore formation has not been reported for this genus, but cell elongation can occur, as observed in the case of carbon limitation. The observed changes in cell morphology may play a role in the degradation of the polymer and/or the shuttling of nutrients between cells on the PS surface, and the question remains as to which genes are affected, other than those involved in the PS degradation pathway.

### 3.4. Chemical Analysis of Biodegraded PS

**Figure 6:**
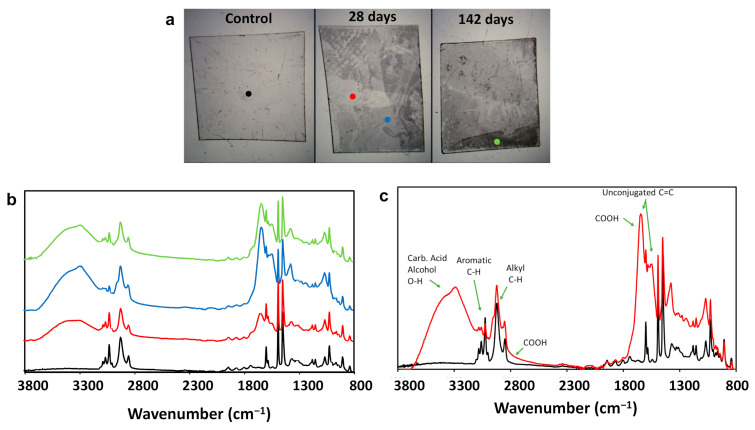
ATR-FTIR analysis of PS degradation after exposure to *Exiguobacterium* sp. RIT 594 for different periods of time. (**a**) Pictures of PS in culture media for 28 days (left) and exposed to the bacterium for either 28 (middle) or 142 (right) days. Color dots represent where ATR-FTIR spectra were obtained. (**b**,**c**) ATR-FTIR spectra of PS after different exposures. (**b**) The spectrum colors correspond to the colored points shown in panel A. PS in the control shows no degradation, being identical to pristine PS. Spectra of PS exposed to the bacterium indicate that the biotransformation occurring in styrene involves the addition of both hydroxyl and carboxylic acid functional groups to the benzyl ring. (**c**) Assignment of additional IR peaks from the degradation of PS by *Exiguobacterium* sp. RIT 594 (red traces) compared to the control sample (black traces) after 28 days of bacterial exposure.

To elucidate the mechanism of degradation by the bacterium, we analyzed samples of PS exposed to *Exiguobacterium* sp. RIT 594 using Fourier transform infrared (FTIR) spectroscopy. In the experiment, one piece of polystyrene was kept in air in a petri dish, one was immersed in the culture media without the bacterium (Figure 6a, control), and another was immersed with the bacterium for either 28 or 142 days (Figure 6a, middle and right). Clear degradation can be observed on the polystyrene exposed to the bacterium. The degradation was not homogenous across the surface, but rather in patches producing a surface with different degrees of degradation. The FTIR spectra of PS in culture medium only (control) or in contact with the bacterium for 28 or 142 days is shown in Figure 6b. The spectrum of PS from the control sample is identical to pristine PS, whereas several new peaks can be observed from the PS exposed to the bacterium. Together, these results provide further confirmation that PS degradation indeed occurs due to the action of *Exiguobacterium* sp. RIT 594.

The changes in the IR peaks provided insights into the biochemical mechanism of degradation. First, there are two sets of peaks for C-H stretches on PS, one from 2800–2980 cm^−1^ and the second from 3000–3125 cm^−1^, which arise from alkyl and aromatic C-H bonds, respectively. As can be seen in Figure 6b,c, the aromatic peaks become smaller as more degradation occurs, indicating that most degradation is occurring at the benzyl ring of PS. In addition, attachment at the benzyl ring is confirmed due to the formation of peaks at ~1450 and 1550 cm^−1^, which is indicative of unconjugated double bonds. Further analysis of the IR spectrum of the degraded PS indicates that alcohol and carboxylic acid functional groups have been added to the styrene scaffold. Two overlapping O-H stretches can be observed between 3200–3650 cm^−1^, presumably one being from carboxylic acid and the other from an alcohol group. Furthermore, a broad peak can be observed beneath the C-H stretches that is most likely from the -COOH group of a carboxylic acid. The strong peak at ~1550 cm^−1^ shows additional evidence of carboxylic acid formation since absorption in this region is highly indicative of carbonyl (C=O) bond vibrations (in addition to that of the unconjugated double bond mentioned above). Collating this information together, we reasoned that the aromatic ring is likely being attacked during the initial catabolic biochemical reactions.

### 3.5. The Effects of Oxygen Deprivation

From the bioinformatics genome analysis, we found two putative enzymes that could be participating in the PS degradation pathway: an orphan aromatic ring-cleaving dioxygenase and a hydrolase. We hypothesized that the former could be involved in the initial attack on the benzene rings of the PS polymer, and the latter in later steps of the pathway. To further study this, we investigated whether the presence of oxygen in the cultures was necessary for biodegradation to occur. When oxygen-limited cultures were incubated with PS pieces for up to 28 days, no degradation was observed since the FTIR spectra resembled those of the control (black traces in Figure 6b,c). Therefore, we concluded that molecular oxygen is essential for PS degradation by *Exiguobacterium* sp. RIT 594, which automatically precluded the anaerobic pathways for PS degradation. Moreover, the IR data showed enough evidence to establish the occurrence of a benzene ring attack and exclude pathways via side chain modification that led to styrene-oxide, 1-phenylethanol, and 2-phenylethanol.

### 3.6. Putative Pathways for PS Degradation

We chose PS for our studies because it is a polymer considered to be recalcitrant in terms of biodegradability. Assuming initial depolymerization of PS to styrene, many bacteria can further degrade this molecule under aerobic conditions [62]. There are four major pathways in this case: (a) the monooxygenase pathway, where styrene is first oxidized to styrene oxide via SMO and transformed in phenylacetaldehyde via isomerization by SIO [13], followed by the degradation of phenylacetaldehyde; (b) the ethylbenzene pathway, by which styrene is reduced to ethylbenzene, followed by further catabolism of the latter compound; (c) the side chain pathway, where styrene’s side chain is hydroxylated to either 1-phenylethanol or 2-phenylethanol [14], which are further degraded; and (d) the ring dioxygenase pathway, in which styrene is first transformed into vinylcatechol through ring dioxygenation, followed by further oxidation reactions [17]. Complete pathways are known for the variants (a), (c), and (d) [63].

Our work is the latest to describe PS degradation by an *Exiguobacterium* strain involving the formation of fibers, the subsequent breakdown into nanoscale particles (as seen in Figure 3 and Figure 4), and the further degradation of styrene particles. The initial biochemical steps in the PS degradation pathway from our strain are currently unknown. A search of the genome for orphan genes capable of aromatic ring oxidation and hydrolysis of the subsequent products returned was performed. After manual curation of the BLAST searches of the resulting hits, a few candidates for monooxygenases, dioxygenases, and hydrolases relevant to PS degradation were identified (Table 1 and Table 2). Most of them had identity or high similarity to enzymes only in other members of the *Exiguobacterium* genus, or related genera among the Firmicutes, such as *Bacillus* (data not shown). Clustal Omega analysis [57] showed that the three annotated ring-cleaving dioxygenases of *Exiguobacterium* sp. RIT 594 were 33–43% identical to each other, while the glyoxalase/extradiol dioxygenase proteins were less similar (17–21% identity) to the ring-cleaving group. Well-characterized proteins encoding styrene mono- or dioxygenases reported from *Pseudomonas* or *Rhodococcus* strains [64] were not found.

**Figure 7:**
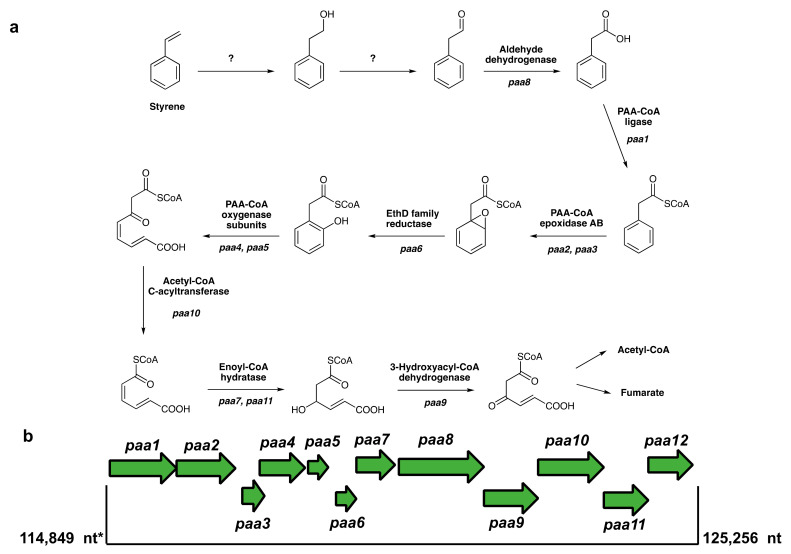
Genome-based pathway prediction for PS degradation *in Exiguobacterium* sp. RIT 594. (**a**) PS is presumably depolymerized to styrene and then metabolized via phenylacetic acid (PAA). The initial steps are unknown (question marks) and the genes corresponding to the enzyme activities predicted from the genome annotations are shown in bold italics. (**b**) The *paa* gene cluster showing the arrangement of genes and the location in the genome (114,849–125,256 nt). * = alternate start at 114,872 nt. *paa1* = phenylacetate-CoA ligase, *paa2* = 1,2-phenylacetyl-CoA epoxidase subunit A, *paa3* = 1,2-phenylacetyl-CoA epoxidase subunit B (paaH), *paa4* = phenylacetate-CoA oxygenase subunit (paaI), *paa5* = phenylacetate-CoA oxygenase subunit (paaJ), *paa6* = EthD family reductase, *paa7* = enoyl-CoA hydratase, *paa8* = aldehyde dehydrogenase, *paa9* = 3-hydroxyacyl-CoA dehydrogenase, *paa10* = acetyl-CoA C-acyltransferase, *paa11* = enoyl-CoA hydratase, and *paa12* = paa degradation operon negative regulatory protein (paaX). The thioesterase is located separately at 114,128–114,523 nt. The gene names in parentheses indicate the nomenclature used for *E. coli* K12. The enzymes for styrene epoxide/phenylethanol generation and oxidation to phenylacetaldehyde are missing (indicated by “?”).

One possibility, therefore, is the pathway shown in Figure 7a, which is suggested by the presence of an entire gene cluster (114,849–125,256 nt) (Figure 7b) in the genome, encoding enzymes for the conversion of phenylacetic acid (PAA) to fumarate and acetyl-CoA. The annotation of our *Exiguobacterium* genome lacks homologs of the oxepin-CoA related isomerase, which is characteristic of the PAA degradation pathway established in *E. coli* K12 [65]. Some of the genes in this cluster are the phenylacetate-CoA oxygenase subunits PaaI (RDB32562.1) and PaaJ (RDB32563.1), the 1,2-phenylacetyl-CoA epoxidase subunits A (RDB32560.1) and B (RDB32561.1), the phenylacetate-CoA ligase (RDB32559.1), and a family 13 thioesterase (RDB32558.1). These are part of the phenylacetyl-CoA catabolon [66], which includes family 13 thioesterase [67]. An unusual putative ether reductase of the EthD family is also present in the gene cluster, suggesting a pathway different from the one involving an oxepin-CoA intermediate. EthD itself is proposed to be a ferredoxin-associated reductase involved in ethyl tertiary butyl ether degradation in *Rhodococcus ruber* [68]. However, an RT-PCR assay to detect the upregulation of the *paa* genes was not conclusive (data not shown). In addition, the genes for the initial enzymes to convert styrene to phenylacetaldehyde, styrene monooxygenase and styrene oxide isomerase, could not be detected in the genome, and the chemical analysis of PS pieces treated with our bacterium in the presence and lack of oxygen (see Section 3.4 and Section 3.5) do not support the hypothesis of degradation via this pathway (Figure 7a).

**Figure 8:**
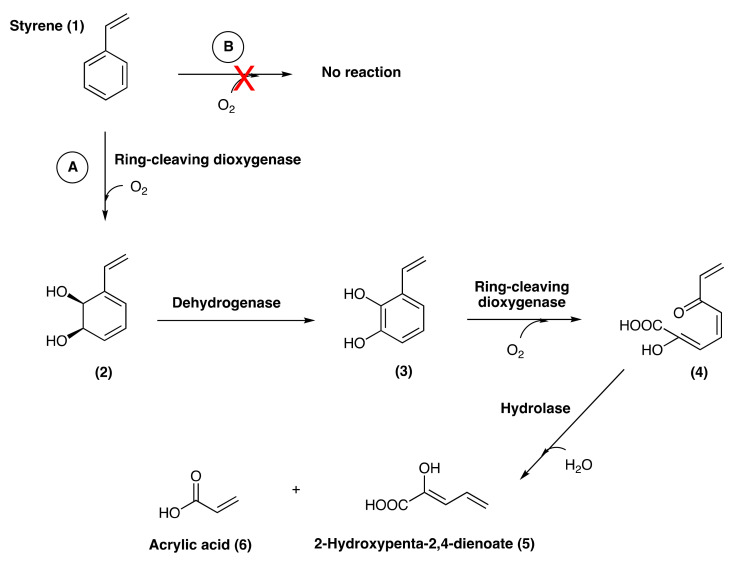
Proposed oxygen-dependent pathway of PS degradation based on the experimental data via activities of an orphan aromatic ring-cleaving dioxygenase and hydrolase enzymes [**A**]. No degradation occurs under oxygen-limited conditions [**B**].

Since neither classical monooxygenases or dioxygenases could be detected in the genome, nor did the identified oxygenases show significant similarity to well-characterized PS degradation enzymes, we postulated that an atypical aromatic ring-cleaving dioxygenase (or an atypical monooxygenase) may be involved in the initial pathway reactions facilitated by the presence of molecular oxygen. This should lead to an initial dearomatization and breakdown of the benzene ring of styrene, and the hydrolase should catalyze the subsequent steps in the degradation of styrene. The second PS biodegradation pathway proposed for our bacterium can be seen in Figure 8, whereby a four-step catalysis by three different enzymes yields C3–C5 compounds. The first step would be the addition of two hydroxyl groups to styrene (**1**) by the ring-cleaving dioxygenase (or 2 rounds of monooxygenase) in the presence of oxygen to generate **2**. Next, a brief aromatization recovery via ring oxidation by a dehydrogenase (**3**) would follow a benzene ring cleavage with O_2_ carried out by the same ring-cleaving dioxygenase enzyme to give **4**. Finally, the hydrolase enzyme would breakdown compound **4** to produce 2-hydroxypenta-2,4-dienoate (**5**) and acrylic acid (**6**). It is worth noting that the second oxidation step does not require a specific enzyme as all genomes have a plethora of dehydrogenases, many of which are known to be promiscuous. Additionally, we propose that the initial ring attack as well as the ring cleavage are caused by the same enzyme, an orphan ring-cleaving dioxygenase. This represents an uncharacterized enzymatic mechanism for PS degradation in the genus *Exiguobacterium*, which resembles the styrene dimer pathway detected using radioactive C^14^-styrene in a soil-derived enrichment culture in 1978 [69].

## 4. Conclusions

In this study, we report the first evidence for a potentially novel oxygen-dependent enzyme for PS biodegradation by *Exiguobacterium* sp. RIT 594, a soil bacterium. Our work shows evidence for a considerable dearomatization of styrene coupled with an increase in non-conjugated C-C double bonds during PS degradation by this strain. Initial vetting of the genome suggested that a potential pathway existent in our strain of *Exiguobacterium* sp. RIT 594 involves an early conversion of styrene to phenylacetate, followed by benzene ring epoxidation. This mechanism resembles the hybrid aerobic pathway that produces phenylacetate from styrene studied by Teufel et al. [65]. Conversely, the *Exiguobacterium* sp. RIT 594 genome does not encode any enzyme with significant sequence similarity to the classical mono- or dioxygenases but possesses several aromatic ring-cleaving dioxygenases and vicinal oxygen chelate (VOC) family enzymes, as well as multiple hydrolases. Because this genus comprises facultative anaerobes [70,71], we reasoned that if molecular oxygen were important to initiate PS degradation, growth under microaerophilic or anaerobic conditions would abolish the degradation activity. On the other hand, if epoxidation or direct hydroxylation were involved, the lack of molecular oxygen would probably not change or affect the PS degradation activity, since bacterial growth can also occur under oxygen-deprived conditions. Our results show that oxygen deprivation indeed abolished PS degradation by *Exiguobacterium* sp. RIT 594 since the incubated PS resembled the bacterium-free control sample. This provides the first evidence for a molecular oxygen-dependent pathway in *Exiguobacterium* sp. likely involving a hitherto uncharacterized dioxygenase enzyme for PS degradation by this genus.

Based on our incubation, genomics and bioinformatics, and spectroscopic studies, we concluded that *Exiguobacterium* sp. RIT 594 degrades PS via biotransformation of styrene monomers into 2-hydroxypenta-2,4-dienoate and acrylic acid. The most parsimonious explanation for the observed IR spectra seems to be direct ring oxygenation. The pattern of the IR signals combined with the presence of a ring-cleaving dioxygenase and a hydroxylase in the genome is most consistent with the pathway shown in Figure 8. This involves an initial hydroxylation and ring cleavage catalyzed by the same dioxygenase enzyme, followed by degradation via hydrolase activity. Overall, we propose this short pathway as a novel route for PS degradation by *Exiguobacterium* sp. RIT 594. Future work will involve determining the identity of the enzymes and clarifying whether other *Exiguobacterium* strains utilize the same pathway to degrade PS.

## Figures and Tables

**Table 1:** Putative orphan proteins involved in PS degradation identified from searches of the genome annotations for *Exiguobacterium* sp. RIT 594. Comparisons of RIT 594 proteins with proteins attributed to styrene degradation in previously characterized *Rhodococcus opacus* [13], -styrene monooxygenases (SMO1 and SMO2), and styrene oxide isomerase (SOI).

Enzyme Annotations	Genome Location (nt)	Protein ID	Identity to *R. opacus* SMO1 (Query Cover)	Identity to *R. opacus* SMO2 (Query Cover)	Identity to *R. opacus* SOI (Query Cover)
Antibiotic biosynthesis monooxygenase	130,054–130,347	RDB32576.1	-	-	-
Heme oxygenase	173,723–174,055	RDB32997.1	-	-	-
Monooxygenase, FAD-dependent monooxygenase	851,364–852,938	RDB33898.1	35% (5%)	-	31.88% (14%)
Antibiotic biosynthesis monooxygenase	1,100,990–1,101,292	RDB34148.1	-	-	-
Antibiotic biosynthesis monooxygenase	130,054–130,347	RDB32576.1	-	-	-
Heme oxygenase	173,723–174,055	RDB32997.1	-	-	-
Monooxygenase, FAD-dependent monooxygenase	851,364–852,938	RDB33898.1	35% (5%)	-	31.88% (14%)
Antibiotic biosynthesis monooxygenase	1,100,990–1,101,292	RDB34148.1	-	-	-
Antibiotic biosynthesis monooxygenase	130,054–130,347	RDB32576.1	-	-	-
Heme oxygenase	173,723–174,055	RDB32997.1	-	-	-

**Table 2:** Putative orphan proteins involved in PS degradation identified from searches of the genome annotations for *Exiguobacterium* sp. RIT 594. Comparisons of RIT 594 proteins with proteins attributed to styrene degradation in previously characterized dioxygenases from *Pseudomonas putida* [18], *Rhodocococus rhodocrous* [17], and *Cupravidus necator* [19]. The hydrolases and reductases can degrade the initial ring-opened products further. The aromatic amino acid hydroxylase is an aromatic ring hydroxylase and could be an unspecific PS depolymerizing enzyme as suggested by other authors [10].

Enzyme Annotations	Genome Location (nt)	Protein ID	Identity to *P. putida* DO (Query Cover)	Identity to *R. rhodocrous* CatA (Query Cover)	Identity to *R. rhodocrous* Extradiol DO (Query Cover)	Identity to *C. necator* DO (Query Cover)
Ring-cleaving dioxygenase	126,220–127,185	RDB32955.1	-	-	26.92% (8%)	-
Ring-cleaving dioxygenase, VOC family protein	139,158–140,096	RDB33200.1	-	-	40.74% (8%)	-
VOC family protein, Glyoxalase, Extradiol dioxygenase, Bleomycin resistance protein	1,198,498–1,198,878	RDB34253.1	-	-	23.40% (14%)	-
VOC family protein, glyoxalase	1,244,862–1,245,542	RDB34313.1	-	-	-	-
Ring-cleaving dioxygenase	1,423,920–1,424,903	RDB34501.1	-	-	26.92% (15%)	-
Alpha/beta hydrolase, Dienelactone hydrolase family protein	1,066,665–1,067,267	WP_158537521.1				
Esterase/lipase	1,422,520–1,423,257	RDB34499.1				
Fumarylacetoacetate (FAA) hydrolase family protein, 2-Keto-4-pentenoate hydratase, 2-oxohepta-3-ene-1,7-dioic acid hydratase (catechol pathway)	1,490,795–1,491,628	RDB34570.1				

## Data Availability

The genome of *Exiguobacterium* sp. RIT 594 has been submitted to the NCBI GenBank under the accession number QPKF00000000.
